# Insulin Resistance Remission Following Laparoscopic Roux-en-Y Gastric Bypass and Laparoscopic Sleeve Gastrectomy in Chinese Type 2 Diabetes Mellitus Patients With a Body Mass Index of 27.5–32.5 kg/m^2^

**DOI:** 10.3389/fphys.2021.772577

**Published:** 2021-11-08

**Authors:** Ping Luo, Yaoquan Cao, Pengzhou Li, Guohui Wang, Zhi Song, Weizheng Li, Zhihong Su, Hui Zhou, Xianhao Yi, Zhibing Fu, Xulong Sun, Haibo Tang, Beibei Cui, Qianqian Yu, Liyong Zhu, Shaihong Zhu

**Affiliations:** Department of General Surgery, Third Xiangya Hospital, Central South University, Changsha, China

**Keywords:** type 2 diabetes mellitus, bariatric surgery, insulin resistance, hyperinsulinemic euglycemic clamp, oral glucose tolerance test

## Abstract

**Background:** Insulin resistance (IR) is closely associated with the pathogenesis of type 2 diabetes mellitus (T2DM). However, remission of insulin sensitivity after bariatric surgery in patients with T2DM and a body mass index (BMI) of 27.5–32.5 kg/m^2^ has not been fully elucidated.

**Methods:** Thirty-six T2DM patients with a BMI of 27.5–32.5 kg/m^2^ were prospectively consecutively recruited for laparoscopic Roux-en-Y gastric bypass (LRYGB) or laparoscopic sleeve gastrectomy (LSG). Hyperinsulinemic euglycemic clamp, oral glucose tolerance test (OGTT), and other indicators were tested at baseline and 6 months postoperative. Glucose disposal rate (GDR), time to reach euglycemia, homeostatic model assessment of IR, quantitative insulin sensitivity check index (QUICKI), triglyceride glucose (TyG) index, 30-min insulinogenic index (IGI30), and disposition index (DI) were calculated at baseline and 6 months after surgery. The criterion for remission in T2DM patients was the achievement of the triple composite endpoint.

**Results:** Anthropometric and glucolipid metabolism parameters significantly improved following surgery. The GDR increased significantly from baseline to 6 months after LRYGB (from 4.28 ± 1.70 mg/kg/min to 8.47 ± 1.89 mg/kg/min, *p* < 0.0001) and LSG (from 3.18 ± 1.36 mg/kg/min to 7.09 ± 1.69 mg/kg/min, *p* < 0.001). The TyG index decreased after surgery (RYGB group, from 9.93 ± 1.03 to 8.60 ± 0.43, *p* < 0.0001; LSG group, from 10.04 ± 0.79 to 8.72 ± 0.65, *p* = 0.0002). There was a significant reduction in the IGI30 (RYGB group, from 2.04 ± 2.12 to 0.83 ± 0.47, *p* = 0.005; LSG group, from 2.12 ± 1.73 to 0.92 ± 0.66, *p* = 0.001). The mean DI significantly increased from 1.14 ± 1.35 to 7.11 ± 4.93 in the RYGB group (*p* = 0.0001) and from 1.25 ± 1.78 to 5.60 ± 4.58 in the LSG group (*p* = 0.003). Compared with baseline, HOMR-IR, QUICKI, area under the curve-C-peptide release test (AUC-CRT), and AUC-OGTT were significantly changed at 6 months postoperative. Overall, 52.63% of patients in the LRYGB group versus 29.41% of patients in the LSG group achieved the triple composite endpoint.

**Conclusion:** Both LRYGB and LSG effectively induced remission of IR in patients with T2DM and a BMI of 27.5–32.5 kg/m^2^.

## Introduction

China had the largest number of adults with diabetes in 2019, a population that is projected to increase to 147.2 million by 2045 (Saeedi et al., 2019). Laparoscopic Roux-en-Y gastric bypass (LRYGB) and laparoscopic sleeve gastrectomy (LSG) are the most commonly performed bariatric procedures and appropriate treatments for patients with type 2 diabetes mellitus (T2DM), especially for those with comorbidities (Zimmet et al., 2011). Bariatric surgery is among the most effective treatments for patients with T2DM and a body mass index (BMI) >35 kg/m^2^ or 30–35 kg/m^2^ with uncontrolled conditions (Zimmet et al., 2011). According to the International Diabetes Federation statement, the BMI threshold for Asian patients with T2DM should be reduced by 2.5 kg/m^2^ (Dixon et al., 2011). Available evidence indicates that surgery is one option, along with lifestyle and medical interventions, for cure in Asian T2DM patients with a BMI <35 kg/m^2^, and that the BMI cut-off point for surgery be lowered for Asian patients with T2DM (Lee and Aung, 2016).

It is well known that insulin resistance (IR) is closely associated with the pathogenesis of T2DM (DeFronzo, 2004). T2DM in the Chinese population is characterized by a low BMI, abdominal obesity, significant IR, and impairment of islet cell function in the early stages of diabetes (Chan et al., 2009). IR is reportedly significantly associated with incident diabetes compared with β-cell dysfunction in the Chinese population, especially in adults with obesity (Wang et al., 2020). Hence, it is critical to accurately assess the IR of patients with T2DM and obesity. The principal methods for evaluating IR in clinical practice are the homeostatic model assessment of IR (HOMA-IR), quantitative insulin sensitivity check index (QUICKI), oral glucose tolerance test (OGTT) with modeling, and hyperinsulinemic euglycemic clamp (Mingrone and Cummings, 2016). The HOMA-IR is the most popular but least accurate and varies among populations (Matthews et al., 1985). The QUICKI is a simple and accurate method for assessing insulin sensitivity in humans (Katz et al., 2000). The triglyceride glucose (TyG) index, as a reliable surrogate marker, has also been used to evaluate IR (Zhang et al., 2017). The clamp is considered the gold standard and is used to evaluate other indicators (Rabasa-Lhoret and Laville, 2001).

Beneficial effects of bariatric surgery on glucose metabolism are known, but the remission of insulin sensitivity after bariatric surgery in T2DM patients with a BMI of 27.5–32.5 kg/m^2^ has not been fully elucidated. This prospective cohort study aimed to use the hyperinsulinemic euglycemic clamp and other indicators to evaluate the effect of LRYGB and LSG on insulin sensitivity for T2DM patients with a BMI in 27.5–32.5 kg/m^2^.

## Materials and Methods

### Study Design and Subjects

Thirty-six T2DM patients with a BMI of 27.5–32.5 kg/m^2^ were prospectively consecutively recruited from July 2019 to June 2020 in the Department of General Surgery, Third Xiangya Hospital, Central South University (Changsha, China). Surgery was safely performed by the same surgical group and the procedure was performed as previously described (Yu et al., 2021). The protocol was approved by the Ethical Committee of our hospital (R19025). Informed consent was obtained from all patients before the study.

The inclusion criteria were as follows: (1) diagnosis of T2DM conforming to the guideline of (Diagnosis and classification of diabetes mellitus, American Diabetes Association, 2014); (2) BMI of 27.5–32.5 kg/m^2^; (3) age 18–65 years; and (4) T2DM duration <15 years. The exclusion criteria were: (1) surgical contraindications to LSG and LRYGB; (2) severe T2DM comorbidities or organic diseases, such as myocardial infarction, renal failure, or stroke; (3) alcohol or medicine addiction; and (4) uncontrolled psychiatric disease.

### Study Protocol and Data Collection

Insulin sensitivity was evaluated at baseline and at 6 months postoperative. Insulin sensitivity was measured using the hyperinsulinemic euglycemic clamp, HOMA-IR, QUICKI, and TyG index. The TyG index was calculated as ln [fasting triglyceride (mg/dL) × fasting glucose (mg/dL)/2]. HOMA-IR was calculated as fasting glucose (mmol/L) × fasting insulin (μU/mL)/22.5. QUICKI was calculated as 1/[log (fasting insulin) + log (fasting glucose)]. Insulin secretion and β-cell function were measured using OGTT modeling. T2DM remission was evaluated using the composite triple endpoint at a 6-month follow-up. The criterion for achieving the triple composite endpoint was a glycosylated hemoglobin A1c (HbA1c) <7.0%, low-density lipoprotein <2.59 mmol/L, and systolic blood pressure <130 mmHg (Ikramuddin et al., 2015).

Anthropometric and glucolipid metabolism parameters were collected at baseline and 6 months postoperative.

### Hyperinsulinemic Euglycemic Clamp

All patients underwent hyperinsulinemic euglycemic clamp experiments at baseline and 6 months after surgery. Catheters were inserted into the antecubital vein for infusion and in the dorsal vein for blood sample collection. A heated box was used on the blood-taking arm to obtain arterialized venous blood. Insulin (Humulin R, Eli Lilly, United States) was administered intravenously at a rate of 40 mU/kg/min for 150 min. Blood samples were drawn using an intravenous catheter in a heated vein, and glucose concentrations were measured at 5-min intervals. Dextrose 20% was infused at variable rates to maintain a glucose level of 5.0 mmol/L. The time taken to reach euglycemia was calculated from the beginning of the experiment to euglycemia. Glucose disposal rate (GDR) was measured during steady-state intervals (Zhao et al., 2017).

### Oral Glucose Tolerance Test

All participants completed the OGTT at baseline and 6 months postoperative. All patients fasted for at least 10 h and ingested 250 mL of water (including 75 g of glucose) in 5 min. Blood samples were collected from the median cubital vein at 0, 30, and 120 min. Based on the OGTT data, we calculated the 30-min insulinogenic index (IGI30) as [C-peptide 30 min (ng/mL) – C-peptide 0 min (ng/mL)]/[glucose 30 min (mmol/L) – glucose 0 min (mmol/L)]. Insulin secretion was assessed using IGI30 and the area under the curve (AUC) for C-peptide release test (AUC-CRT). Disposition index (DI), calculated by multiplying the GDR by IGI30, was used to evaluate β-cell function (Lee et al., 2017; Lemieux et al., 2019).

### Statistical Analysis

The data analysis was performed using SPSS software version 26 (SPSS Inc.). Continuous variables are expressed as mean ± standard deviation. Categorical variables are expressed as frequencies and percentages. The normality of the continuous variables was evaluated using the Shapiro–Wilk test. The normality distribution variables were compared using the Wilcoxon signed rank or Student’s paired *t*-test for baseline and postoperative data. Non-normally distributed variables were compared using the non-parametric test. Categorical variables were compared using the chi-square test. Statistical significance was defined as *p* values < 0.05.

## Results

### Subject Characteristics

A total of 36 patients (17 in the LSG group and 19 in the LRYGB group) with T2DM and a BMI of 27.5–32.5 kg/m^2^ participated in this study. All participants completed the surgery and follow-up. Accordingly, data from 17 participants (12 men, five women) in the LSG group (mean age, 39.06 ± 13.66 years; mean duration of T2DM, 5.71 ± 4.21 years) and 19 participants (15 men, four women) in the LRYGB group (mean age, 42.53 ± 10.63 years; mean duration of T2DM, 5.68 ± 3.27 years) were analyzed before and 6 months following surgery. Five patients in the LSG group versus nine in the LRYGB group were using insulin. The mean duration of insulin infusion was 1.88 ± 3.41 years in the LSG group and 2.56 ± 3.70 years in the LRYGB group. Eleven patients with hypertension in the LSG group versus 12 patients with hypertension in the LRYGB group were included. In the LSG group, nine patients were treated with calcium channel blockers, and two did not take any antihypertensive medications. In the LRYGB group, eight patients were treated with calcium channel blockers, three with angiotensin-converting enzymes, and one with a calcium channel blocker plus angiotensin-converting enzyme. The other baseline characteristics are shown in Table 1. No significant intergroup differences were found in the characteristics of the subjects.

**TABLE 1:** Comparison of clinical characteristics in the LRYGB and LSG group.

**Characteristics**	**LRYGB (*n* = 19)**	**LSG (*n* = 17)**	***P* value**
Gender	19	17	0.5761
Men	15	12	
Female	4	5	
Age (year)	42.53 ± 10.63	39.0 ± 13.66	0.3987
Duration of T2DM (year)	5.68 ± 3.27	5.71 ± 4.21	0.9863
Metformin	15	10	0.2013
Duration (year)	4.58 ± 3.96	4.24 ± 4.84	0.8164
Insulin infusions	9	5	0.2830
Duration (year)	2.56 ± 3.70	1.88 ± 3.41	0.5623
Comorbidities			
Hypertension	12	11	0.9258
Non-alcoholic fatty liver	15	16	0.1994

As shown in Table 2, BMI, waist-to-height ratio (WtHR), systolic blood pressure, diastolic blood pressure, and triglycerides (TG) decreased significantly at 6 months postoperative versus baseline. The mean fasting glucose decreased from 9.77 ± 3.39 mmol/L to 5.98 ± 1.58 mmol/L in the LSG group versus from 10.16 ± 4.73 mmol/L to 5.68 ± 1.55 mmol/L in the LRYGB group. The mean HbA1c decreased from 8.74 ± 1.77% to 6.02 ± 0.82% in the LSG group and from 8.81 ± 1.93% to 6.1 ± 0.87% in the LRYGB group.

**TABLE 2:** Variables comparison of the LRYGB and LSG group at baseline and post-surgery.

	**LRYGB (*n* = 19)**			**LSG (*n* = 17)**		
	**Basal**	**Post-surgery**	***P* value**	**Basal**	**Post-surgery**	***P* value**
Body mass (kg)	79.479.70	69.4811.52	< 0.0001^****^	89.619.15	79.1210.71	< 0.0001^****^
BMI (kg/m^2^)	28.771.64	25.093.02	< 0.0001^****^	31.251.55	27.512.74	< 0.0001^****^
Waist circumference (cm)	97.506.47	83.638.27	< 0.0001^****^	112.415.37	92.9711.63	< 0.0001^****^
Hip circumference (cm)	99.976.14	91.845.73	< 0.0001^****^	110.810.38	100.88.05	< 0.0001^****^
WHR	0.980.04	0.910.05	< 0.0001^****^	1.010.08	0.920.08	0.0003
WtHR	0.590.38	0.500.04	< 0.0001^****^	0.670.09	0.550.07	< 0.0001^****^
SBP (mmHg)	134.816.98	12321.04	0.0212^*^	135.418.52	125.117.41	0.0764^**^
DBP (mmHg)	86.4712.59	79.2113.55	0.0356^*^	92.9414.74	81.767.46	0.0063^**^
ALT (U/L)	54.5851.54	27.8425.36	0.0556	52.8845.94	23.9419.86	0.0081^**^
AST (U/L)	31.5817.64	24.849.51	0.1806	29.5918.81	19.8810.73	0.0418^*^
LDL (mmol/L)	2.490.86	2.430.38	0.7665	2.560.96	3.020.98	0.1055
HDL (mmol/L)	1.140.24	1.210.23	0.3035	1.010.28	1.160.20	0.0116^*^
TC (mmol/L)	5.150.93	4.390.60	< 0.0001^****^	5.271.16	5.270.90	0.9875
TG (mmol/L)	3.824.10	1.280.44	0.0096^**^	4.104.23	1.561.11	0.0219^*^
Fasting						
Glucose (mmol/L)	10.164.73	5.681.55	< 0.0001^****^	9.773.39	5.981.58	0.0003^***^
HbA1c (%)	8.811.93	6.10.87	< 0.0001^****^	8.741.77	6.020.82	< 0.0001^****^
Hyperinsulinemic clamp						
Glucose disposal rate (mg/kg/min)	4.281.70	8.471.89	< 0.0001^****^	3.181.36	7.091.69	< 0.0001^****^
Time to reach euglycemia (min)	124.49.44	108.113.13	< 0.0001^****^	126.09.61	106.713.82	< 0.0001^****^
OGTT (mmol/L)						
0 min	9.854.69	5.541.20	0.0008^***^	9.322.63	6.212.37	0.0041^**^
30 min	14.053.55	13.623.24	0.6828	13.652.56	12.623.94	0.4028
120 min	17.904.29	7.704.12	< 0.0001^****^	16.993.22	9.254.97	< 0.0001^****^
AUC (min × mmol/L)	1796443.9	1247347.9	< 0.0001^****^	1723307.0	1266417.4	0.0024^**^
OGTT-CRT (μg/L)						
0 min	2.630.88	1.700.46	< 0.0001^****^	3.511.59	3.081.34	0.2304
30 min	3.971.25	7.822.55	< 0.0001^****^	4.782.49	9.306.15	0.0040^**^
120 min	6.162.28	7.403.78	0.2187	8.154.52	8.893.49	0.5365
AUC (min × μg/L)	554.4166.7	827.3277.6	0.0017^**^	706.3362.6	1004493.3	0.0136^*^
HOMA-IR	5.483.86	1.340.72	< 0.0001^****^	10.987.68	4.613.19	0.0004^***^
IGI30	2.042.12	0.830.47	0.005^**^	2.121.73	0.920.66	0.001^**^
Disposition index	1.141.35	7.114.93	0.000^****^	1.251.78	5.604.58	0.004^**^
Triglyceride glucose index	9.931.03	8.600.43	< 0.0001^*^	10.040.79	8.720.65	0.0002^***^
QUICKI	0.520.09	0.740.17	< 0.0001^****^	0.440.06	0.540.09	< 0.0001^****^

*P < 0.05; **P < 0.01; ***P < 0.001; ****P < 0.0001.

*WHR, waist-hip ratio; WtHR, waist-to-height ratio; SBP, systolic blood pressure; DBP, diastolic blood pressure; LDL, low-density lipoprotein; HDL, high-density lipoprotein; TC, total cholesterol; TG, triglyceride; HbA1c, glycosylated hemoglobin A1c; OGTT, oral glucose tolerance test; AUC, area under the curve; CRT, C peptide release test; IGI30, 30-min insulinogenic index; QUICKI, quantitative insulin sensitivity check index.*

### Insulin Sensitivity, Insulin Secretion, and β-Cell Function

As shown in Tables 2, 3, the GDR significantly increased at 6 months after LSG (from 3.18 ± 1.36 mg/kg/min to 7.09 ± 1.69 mg/kg/min) and LRYGB (from 4.28 ± 1.70 mg/kg/min to 8.47 ± 1.89 mg/kg/min). Time to reach euglycemia decreased remarkably from 126.0 ± 9.61 min to 106.7 ± 13.82 min in the LSG group and from 124.4 ± 9.44 min to 108.1 ± 13.13 min in the LRYGB group at 6 months postoperative. The mean QUICKI increased at 6 months after LSG (from 0.44 ± 0.06 to 0.54 ± 0.09) and LRYGB (from 0.52 ± 0.09 to 0.74 ± 0.17). The mean TyG index significantly decreased by 1.32 ± 1.15 in the LSG group and by 1.33 ± 1.11 in the LRYGB group at 6 months postoperative. Meanwhile, a significant change was observed in insulin secretion and β-cell function after bariatric surgery. The mean IGI30 decreased by 1.20 ± 1.14 in the LSG group and by 1.22 ± 1.68 in the LRYGB group. The mean DI increased by 4.29 ± 4.88 in the LSG group and by 5.97 ± 5.09 in the LRYGB group. The AUC-CRT significantly increased 6 months after surgery. Compared with the baseline, other indicators, including HOMR-IR and AUC-OGTT, significantly decreased postoperatively.

**TABLE 3:** Comparison of variable differences of the LRYGB and LSG group between baseline and post-surgery.

	**Bariatric surgery**	
**Variables**	**LRYGB (*n* = 19)**	**LSG (*n* = 17)**
ΔBMI (kg/m^2^)	−3.682.05	−3.731.88
ΔWHR	−0.070.06	−0.090.08
ΔWtHR	−0.090.04	−0.120.05
Fasting		
ΔGlucose (mmol/L)	−4.483.93	−3.793.41
ΔHbA1c (%)	−2.711.78	−2.721.97
Hyperinsulinemic clamp		
ΔGlucose disposal rate (mg/kg/min)	4.191.94	3.901.45
ΔTime to reach euglycemia (min)	−16.3211.88	−19.2910.61
OGTT		
ΔAUC-OGTT (min × mmol/L)	−549.6491.4	−456.7524.5
ΔAUC-CRT (min × μg/L)	272.8323.6	297.8442.8
ΔHOMA-IR	−4.143.72	−6.375.90
ΔIGI30	−1.221.68	−1.201.14
ΔDisposition index	5.975.09	4.294.88
ΔTriglyceride glucose index	−1.331.11	−1.321.15
ΔQUICKI	0.210.18	0.090.06

*WHR, waist-hip ratio; WtHR, waist-to-height ratio; SBP, systolic blood pressure; DBP, diastolic blood pressure; LDL, low-density lipoprotein; HDL, high-density lipoprotein; TC, total cholesterol; TG, triglyceride; HbA1c, glycosylated hemoglobin A1c; OGTT, oral glucose tolerance test; AUC, area under the curve; CRT, C peptide release test; IGI30, 30-min insulinogenic index; QUICKI, quantitative insulin sensitivity check index.*

### Composite Triple Endpoint

As shown in Table 4, the proportion of composite triple endpoint remissions appeared higher in the LRYGB group than in the LSG group (52.63% vs. 29.41%), but the difference was not statistically significant [odds ratio, 0.3750; 95% confidence interval (CI), 0.09252–1.511; *p* = 0.1922].

**TABLE 4:** Comparison of diabetes remission in the LRYGB and LSG group.

**Diabetes remission**	**LRYGB (*n* = 19)**	**LSG (*n* = 17)**	**Odds ratio (95% confidence interval)**	***P* value**
Composite triple endpoint	10 (52.8%)	5 (29.41%)	0.38 (0.09–1.51)	0.1993
Fail to composite triple endpoint	9 (47.37%)	12 (70.59%)		

## Discussion

Type 2 diabetes mellitus is a global problem, but more than 50% of patients are unable to achieve their therapeutic targets (Saydah et al., 2004). IR is an important factor in the pathophysiology of T2DM and it significantly increases the incidence of comorbidities (Way et al., 2016). Bariatric surgery likely contributes to antidiabetic effects through weight loss and other weight-independent mechanisms (Batterham and Cummings, 2016). Previous research has shown that LRYGB and LSG are effective at achieving IR normalization based on the data of patients with severe obesity (Benaiges et al., 2013). However, the data on bariatric surgery for IR remission are insufficient in patients with T2DM and a low BMI.

Here we investigated whether LSG and LRYGB improve insulin sensitivity in patients with T2DM and a BMI of 27.5–32.5 kg/m^2^. No intergroup differences were observed in the baseline characteristics of the patients. To this end, we assessed the GDR, time to reach euglycemia, HOMR-IR, QUICKI, OGTT with modeling, and TyG index. Our data show that, after weight loss induced by LSG or LRYGB, there were significant improvements in the GDR, HOMR-IR, QUICKI, and TyG index. All of the indicators evaluating the IR significantly improved from baseline to 6 months postoperative. Our previous study also showed that IR significantly improved at 3 months following LRYGB in patients with T2DM and a BMI <32.5 kg/m^2^ (Zhao et al., 2017). Similar to our study, a dramatic reduction in IR was observed in patients with T2DM and a BMI of 23–35 kg/m^2^ after LRYGB (Lee et al., 2011). Lee et al. (2010) found that IR decreased in patients with T2DM and a BMI of 25–35 kg/m^2^ following LSG.

A reduction in insulin secretion (Δinsulin/Δglucose in 30 min) and an increase in total insulin secretion (AUC for insulin) was observed at 6 months after bariatric surgery in T2DM patients with a BMI <35 kg/m^2^ (Lee et al., 2011). Similar results for insulin secretion were found using IGI30 and AUC-CRT in our study population. An increase in β-cell mass through “nesidioblastosis” may account for the increase in insulin secretion (Kaiser, 2005). The increase in the insulin secretion rate response to the OGTT, together with the restoration of the first-phase insulin secretion, might explain the reversal of T2DM after LRYGB (Salinari et al., 2013). Early improvement of β-cell function was associated with T2DM remission after LSG (Mullally et al., 2019). Our data showed a significant increase in DI at 6 months postoperative in both the groups. This showed that LRYGB and LSG significantly improved β-cell function, which may be one of the mechanisms of diabetes remission after bariatric surgery.

Meanwhile, other indicators, including BMI, WtHR, blood pressure, TG, fasting glucose, and HbA1c, also significantly improved from baseline to 6 months postoperative in our study groups. Based on our data, we may conclude that LSG and LRYGB are also effective at alleviating IR in Chinese patients with T2DM and a BMI of 27.5–32.5 kg/m^2^.

Both LSG and LRYGB are proven effective options for T2DM and comorbidities, especially in patients with a BMI >35 kg/m^2^ (Zimmet et al., 2011). Our previous studies also demonstrated that weight, glycemic, and lipid profiles improved in T2DM patients with a BMI <32.5 kg/m^2^ who underwent bariatric surgery (Ji et al., 2020). In the present study, anthropometric and glycolipid parameters improved from baseline to 6 months after LSG and LRYGB. This finding is consistent with the results of previous studies (Di et al., 2016; Du et al., 2017; Ji et al., 2020). Consequently, the two bariatric procedures were available for Chinese patients with T2DM and a BMI of 27.5–35 kg/m^2^. Two randomized clinical trials (RCTs) showed no difference in remission of T2DM, quality of life, or individual burden for patients with surgical complications at 5 years postoperative in patients with a BMI >35 kg/m^2^ (Peterli et al., 2017). Based on our results, the proportion of patients achieving composite triple endpoint remission in the LRYGB group seemed higher than that in the LSG group, but the difference was not statistically significant. This may indicate that LRYGB also has no short-term advantage in the improvement of the composite triple endpoint for Chinese patients with T2DM and a low BMI.

The present study had several limitations. First, its sample size was small. Second, the patients were not randomly assigned to the bariatric procedures. Third, the follow-up time was significantly insufficient, and the triple composite endpoint to evaluate the remission of T2DM was not ideal.

## Conclusion

Both LRYGB and LSG effectively achieve remission of IR in patients with T2DM and a BMI of 27.5–37.5 kg/m^2^. Larger sample and longer follow-up RCTs are required to validate our findings.

## Data Availability Statement

The raw data supporting the conclusions of this article will be made available by the authors, without undue reservation.

## Ethics Statement

The studies involving human participants were reviewed and approved by the Human Ethics Committee of the Third Xiangya Hospital of Central South University. The patients/participants provided their written informed consent to participate in this study.

## Author Contributions

PL and YC contributed to study design and writing. PZL, GW, ZS, and WL contributed to surgery. ZHS, HZ, XY, ZF, XS, HT, BC, and QY contributed to data collections and data analysis. LZ and SZ contributed to guide. All authors contributed to the article and approved the submitted version.

## Conflict of Interest

The authors declare that the research was conducted in the absence of any commercial or financial relationships that could be construed as a potential conflict of interest.

## Publisher’s Note

All claims expressed in this article are solely those of the authors and do not necessarily represent those of their affiliated organizations, or those of the publisher, the editors and the reviewers. Any product that may be evaluated in this article, or claim that may be made by its manufacturer, is not guaranteed or endorsed by the publisher.
